# The Perception of Pelvic Floor Muscle Function amongst Exercising Women Who Are Repeatedly Instructed to Contract Their Pelvic Floor Muscles

**DOI:** 10.3390/healthcare10091768

**Published:** 2022-09-14

**Authors:** Gali Dar, Tamar Sharon Saban

**Affiliations:** 1 Department of Physical Therapy, Faculty of Social Welfare & Health Sciences, University of Haifa, Mount Carmel, Haifa 3498838, Israel; 2 Physical Therapy Clinic, The Ribstein Center for Sport Medicine Sciences and Research, Wingate Institute, Netanya 4290200, Israel

**Keywords:** urinary incontinence, pelvic diaphragm, women’s health

## Abstract

In this study, the self-perception of pelvic floor muscle (PFM) contractions amongst women receiving repeated verbal instructions during exercise classes was examined. The prevalence and severity of urinary stress incontinence were also assessed. This cross-sectional observational study included 46 women (mean age 48 (±8.6)), who regularly participated in Pilates classes where repeated instruction was given to contract PFM (“instruction group”; N = 22) or not (controls, N = 24). PFM function was evaluated using transabdominal ultrasound. Simultaneously, the participant described her personal evaluation of her PFM contraction ability. The International Consultation on Incontinence Questionnaire–Short Form was also utilized. Most women (80%) correctly contract PFM; however, 95% did not perform a voluntary contraction during leg movement, without differences observed between groups. A higher perception of PFM contraction was found in the “instruction group” when performing knee flexion towards the chest without specific verbal instruction. Women who were instructed to contract their PFM suffered less incontinence and had a lower degree of severity than the controls. Most women performing Pilates exercises correctly contracted their PFM. However, there was no PFM voluntary contraction during leg movement. Exposure to repeated verbal instructions to contract PFM, over time, might lead to an improvement in women’s perception of their ability to contract PFM. Verbal instructions for PFM contraction were found to be effective in reducing urinary incontinence.

## 1. Introduction

Many women suffer from urinary stress incontinence and pelvic organ prolapse. These conditions produce emotional, medical, and social problems in addition to economic costs [1] and a decreased quality of life [2,3].

The recommended treatments for these disorders resulting from pelvic floor defects in women are divided into two treatment modalities: conservative and surgical [4]. As one of the main reasons for these disorders and symptoms is weakness of the pelvic floor muscles (PFM), the proposed treatment is the strengthening of these muscles [5,6]. Conservative treatment also includes recommendations for lifestyle changes, the use of pelvic floor support accessories, and the use of electrical stimuli [4,5,7]. PFM practice has been found to be effective in treating stress urinary incontinence and pelvic prolapse [8,9,10,11,12], with no side effects or risk [1,11], and was also found to be an effective program for treating urinary incontinence during pregnancy and/or the postpartum period [13,14,15]. 

The goals of PFM exercises are: (a) to learn how to voluntarily perform a muscle contraction before and during an increase in intra-abdominal pressure (before/during coughing, sneezing, lifting weights, jumping, etc.), thus preventing leakage; (b) to strengthen the PFM, which provides support for the pelvic organs and improves the urinary sphincteric closure system [8,16].

Several studies have shown that, following exercise, a change in muscle strength, thickness, and length, as well as an improvement in rectal and bladder position, occurs. Moreover, in women who performed pelvic floor exercises, an automatic contraction of the muscles was observed; hence, exercise appears to not only affect the strength of the muscles, but also improve their neural function [16,17,18]. However, many women do not have the knowledge of how to voluntarily contract the PFM, cannot discern if there has been a contraction, do not contract the muscles correctly, or do not use contraction during actions that cause a strain on the pelvic floor. An incorrect contraction might include a lack of a contraction; the pushing of the pelvic organs and urinary bladder in a downward movement; using other muscles—i.e., abdominal or gluteus; and holding one’s breath [19,20,21,22]. The use of these unnecessary movements while trying to contract the PFM accentuates the difficulty experienced by some women in understanding the function of these muscles. Supervision, instructions, and feedback regarding PFM contraction during exercises might further increase the ability to correctly contract the PFM [1,20].

The aim of this study was to examine the self-perception of PFM contractions amongst women who received repeated verbal instructions during exercise classes compared to that of women who did not receive such instructions and to compare the prevalence and severity of urinary stress incontinence amongst the research groups. We hypothesized that a higher self-perception of PFM contractions would be noted amongst women who repeatedly receive instruction on how to contract their PFM during exercise.

## 2. Materials and Methods

### 2.1. Design

This study was a cross-sectional observational study approved by the University of Haifa’s Institutional Review Board. All the participants signed an informed consent form prior to their participation. The rights of the subjects were protected.

### 2.2. Participants

The study included 46 healthy women (22 in the research (instruction) group and 24 in the controls), aged 30–65, who had previously given birth (at least 1 year after birth), and who had participated in a Pilates exercise group for over a year. Inclusion criteria were regular attendance at a Pilates exercise group for over a year, at least once a week. Exclusion criteria were current treatment with rehabilitation or the practice of PFM, previous abdominal and/or pelvic surgery (excluding a caesarean section), and pregnancy.

The participants were recruited from several Pilates exercise classes. Participants of the “instruction group” were recruited from classes in which verbal instructions to contract their PFM were given at least 4 times during each lesson; the participants in the “control group” were recruited from classes in which PFM was not mentioned during the lessons. Interviews with the Pilates teachers were performed prior to research initialization. In addition, observations of several lessons for each teacher were conducted in order to understand and evaluate the following: common exercises, including instruction for PFM contraction; the exact words of the instruction for carrying out PFM; and frequency of instructions for PFM contraction during each lesson. Individuals attending Pilates classes which included similar exercises participated in this study.

### 2.3. Research Procedure

Each participant completed a demographic characteristics questionnaire (age, height, weight, number of children, physical activity, PFM exercises) and the International Consultation on Incontinence Questionnaire – Short Form, which assesses the presence of urinary incontinence, its severity (frequency and amount of urine lost), and its effect on quality of life. Scoring results range from 0 to 21, with a higher score indicating a greater severity of urinary incontinence [4,23]. 

**Figure 1:**
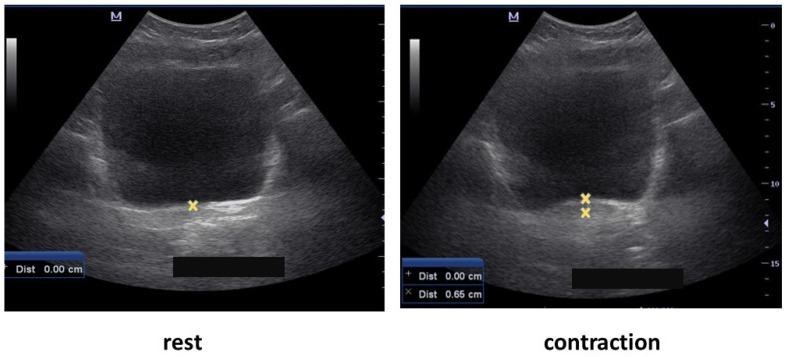
Ultrasound assessment of pelvic floor muscle function: Displacement during rest and contraction.

Following this, an ultrasound examination of the PFM was performed by an experienced physical therapist specializing in pelvic floor disorders using a 6 MHz 35 mm curved linear array ultrasound transducer (Mindray M5). An accepted bladder filling protocol was employed prior to examination to ensure that there was enough fluid in the bladder. The examination was performed in a crook lying position. The transducer was placed in the transverse plane suprapubically over the lower abdomen, and angled at 15–30° from the vertical line. The bladder base was marked on rest with the on-screen marker. The participant was asked to contract her PFM and the bladder base was marked again [24,25]. The direction of the bladder base displacement was recorded, as this indicated PFM function (Figure 1). The upward displacement of the bladder indicated a correct function. No movement indicated a lack of contraction, and a downward movement indicated a wrong contraction and increased intra-abdominal pressure.

The ultrasound examination was performed under three crook lying situations in a fixed order: (a) during a PFM contraction following verbal instructions; (b) during crook lying and lifting the right knee towards the chest (up to a 90° bend in the thigh) without instructions given to contract PFM; and (c) while lifting the right knee towards the chest (up to a 90° bend in the thigh) in combination with a verbal instruction for PFM contraction. The participants performed three contractions of each maneuver. The verbal instructions regarding obtaining PFM contraction were “Squeeze your pelvic floor muscles”. If instruction was given (condition a,c), the participants were asked to perform the contraction prior to leg movement and maintain it throughout the whole movement.

Simultaneously, for each contraction, the participants were asked to provide their personal assessment of their ability to contract their PFM correctly according to two possible groupings: contracted and non-contracted. The objective PFM function and the subjective assessment were recorded and compared.

### 2.4. Data Analysis

Statistical analysis was performed using SAS for Windows, version 9.4. Descriptive statistics were calculated for demographic and participant characteristics. The data were first analyzed to evaluate the normal or non-normal distribution of all demographic parameters and outcome measurements. Chi-square tests were performed in both research groups to examine the relationship between receiving repeated verbal instructions for contracting PFM and PFM function, the incidence of urinary incontinence, and the participant’s perception of the contraction. To assess differences in demographic and outcome measurements between groups, an independent *t*-test and Wilcoxon rank-sum tests were performed for data with normal and non-normal distributions, respectively. In addition, Spearman and chi-square tests were conducted to examine additional possible relationships between the research indices between and within the groups. The alpha level for all measurements was set at *p* < 0.05. The sample size was calculated prior to data collection using the G-Power software to obtain a statistical power of 80% at an alpha level of 0.05 and with an effect size of 0.75, considering the generic comparison of differences between independent means (two groups).

## 3. Results

In total, 46 female participants were enrolled in the study: 22 were in the “instruction group” and there were 24 controls. Descriptive statistics are provided in Table 1. No significant differences were found in the demographic variables between groups (*p* > 0.05). Most of the study participants reported that they had been exercising in their group for >3 years (76%), with no difference found between the groups. All participants reported that they had heard of PFM in the past, and the majority (84.78%) reported that they contracted their PFM during exercise classes, without differences between groups (*p* = 0.34). In contrast, only one-third of the participants reported that they practiced PFM exercises outside of class (30.43%), without differences between research groups (*p* = 0.66).

### 3.1. The Ability to Correctly Contract PFM

The correct contraction of the PFM was determined by the upward movement of the bladder. Most of the study participants (82% and 79% in the “instruction group” and controls, respectively) performed a correct PFM contraction when given verbal instructions while lying in a crook position. None of the participants performed a downward movement of the bladder. No significant difference between research groups was found (*p* = 0.82).

### 3.2. Pelvic Floor Muscle Function during Movement

The function of PFM during the flexion of the right knee towards the chest was assessed with and without verbal instruction for PFM contraction (Table 2). While performing this movement *without* specific verbal instructions to contract the PFM, only one participant in each group performed a correct PFM contraction and maintained the contraction throughout the movement. Two participants in the “instruction group” performed a correct contraction; however, they did not succeed in maintaining the contraction. The vast majority of the study participants (95%) did not correctly contract their PFM: 11 (50%) and 17 (61%) in the “instruction” and “control” groups, respectively, experienced no movement of the bladder base, while 36% and 25% in the “instruction” and “control” groups, respectively, executed the pushing of the bladder in a downward movement. No significant differences between groups were found (*p* = 0.32).

While performing this movement *with* specific verbal instruction to contract their PFM, 26% of the entire sample performed a correct contraction. In total, 43.5% succeeded in performing correct contraction; however, the contraction was released immediately. Furthermore, 19.6% did not perform any contraction (no bladder movement) and 10.9% pushed the bladder in a downward movement. No significant differences between research groups were found (*p* = 0.17).

### 3.3. Self-Perception of A PFM Contraction

The participants’ perception of a PFM contraction was evaluated by examining the agreement between the subjective reporting of the participants and the objective assessment of a PFM contraction in each position tested. Subjective reporting was determined by each participant’s personal report, whether she felt that she was contracting her PFM or not. The objective assessment relates to whether an upward displacement of the bladder base, considered as a correct movement, exists during ultrasound examination. The results are summarized in Table 3. In most of the study participants (80.4%), an agreement was found between the subjective reporting and the objective examination of a PFM contraction while lying in a crooked position and given verbal instruction. No significant differences between research groups were found (*p* = 0.82).

While performing a movement consisting of knee flexion towards the chest *without* specific verbal instruction for a PFM contraction, a significant difference between groups was found. In 20 (91%) and 17 (71%) participants in the “instruction group” and controls, respectively, an agreement between the subjective reporting and the objective examination was found (*p* = 0.027). A higher prevalence of agreement was found in the “instruction group”, thus implying a greater perception of a PFM contraction.

While performing the movement of the knee flexion towards the chest *with* specific verbal instruction for PFM contraction, a significant difference was found between groups (*p* = 0.02). In 5 (23%) “instruction group” participants and 13 (54%) controls, an agreement was found. Moreover, in 12 (55%)”instruction group” participants compared with 4 (17%) controls, a partial agreement was found, implying that the participant reported that she felt a PFM contraction; however, in the objective examination conducted via ultrasound, the contraction was viewed as a short contraction and fast release. Thus, in 5 (23%) and 7 (29%) participants in the “instruction” and control groups, respectively, no agreement at all was found. When combining agreement and partial agreement situations together, no differences between groups were found to exist. During PFM contraction without verbal instruction, both groups had a better perception compared with the verbal instruction condition.

### 3.4. Urinary Incontinence

The presence of urinary incontinence and its severity were assessed by the International Consultation on Incontinence Questionnaire – Short Form. In the “instruction group”, 16 women (73%) reported that they had not experienced urinary incontinence compared with 10 women (42%) in the controls (*p* < 0.034); 4 (18%) reported weak incontinence (score 1–5) compared with 6 (25%) in the controls (*p* = 0.57); and 2 (9%) reported moderate incontinence (score 6–12) compared with 8 (33%) in the controls (*p* = 0.04). The highest score amongst the “instruction group” was 6 compared with 12 in the controls, thus implying that women who receive instructions to contract their PFM during exercise suffer less from urinary incontinence or severe symptoms compared with women who do not receive such instructions.

## 4. Discussion

Practicing and strengthening PFM is the first line of treatment and a major part of the treatment offered for PFM disorders, such as stress urinary incontinence and pelvic organ prolapse [4,5,7,9]. These conditions are common in women, and their incidence increases as age increases [26]. Nevertheless, some women do not know how to contract their PFM proactively or contract them correctly [1,19,20,21,22,27].

The current study examined whether exposure to repeated verbal instructions to contract PFM over time would lead to an improvement in the women’s perception of their ability to contract their PFM and to actually improve their ability to correctly contract their PFM. In addition, we examined whether exposure to repeated verbal instructions to contract PFM over time was associated with a lower incidence of urinary leakage. The study was conducted amongst a population of women who performed Pilates exercises, since repeated verbal instructions are given to contract PFM only in some Pilates groups. Thus, it was possible to conduct a cross-sectional study of a population of women who had previously been exposed to repeated instructions to contract their PFM and compare them to a similar population who had not been exposed to such instructions.

The main results of this study show that long-term exposure to instructions to carry out PFM contractions is associated with a lower incidence and severity of urinary incontinence and with a slight improvement in women’s perception of their ability to contract their PFM. However, it is unrelated to the ability to correctly contract their PFM. The percentage of women who managed to correctly contract their PFM was 80.4%; 19.6% did not know how to properly contract their PFM at all in a crook lying position. These findings are similar to the results found in previous studies [1,20,22] and in partial contrast to those found other studies, which showed worse outcomes [21,28]. The difference between the studies stems from the various study populations (age, number of births, symptoms, etc.) and different measurement tools (manual examination, observation, perineal or abdominal ultrasound, etc.) used.

The population of the present study is very similar to that of Barton et al. [29], who reported on women who participated in a group exercise program using the same measurement tools as those in our study (2D abdominal ultrasound). The authors also found a similar percentage of participants who correctly contracted their PFM (75%) in a crook lying position. Moreover, during a curl-up exercise, they found that all women displayed bladder-base depression. In the present study, 30.4% of the participants pushed the bladder downwards when asked to contract their PFM while lying in a crook position with a raised leg; however, 60% did not perform any bladder movement. Despite the similarities in the characteristics of the participants in both studies, it should be noted that the study population of Barton et al. [29] included women from different types of sports groups, including high-intensity sports—i.e., running, ball games, and aerobic exercise—whereas the present study was conducted only on women performing Pilates exercises. Pilates largely avoids high impacts, high power outputs, and heavy muscular and skeletal loading.

In the present study, the function of PFM while flexing the knee towards the chest as a distal movement was examined. Most of the women in both groups (90%) did not contract their PFM while performing leg movements unless explicitly instructed to do so, thus reducing the percentage to 73%. These findings are consistent with those of Baessler and Junginger [30] for women aged 35–45 without pelvic floor disorders while performing various Pilates exercises. Although all the women were able to perform voluntary pelvic floor contractions, not all did so while performing the exercises. Some pushed the bladder down and 50% had difficulty in maintaining a contraction during the various practice movements, which improved with specific instructions.

The majority of our participants (80%) accurately reported a self-perception of PFM contraction evaluated based on the agreement between the subjective reporting and the objective examination of a PFM contraction when performed with verbal instruction in a crook lying position. This level of agreement was lower when performing a leg raise without instructions and the lowest while performing a leg raise with instructions. As self-perception also refers to there being no contraction taking place and as most of the women did not contract their PFM while performing leg movements, it might be that, during leg movement without verbal instruction, women felt more comfortable declaring that they felt no contractions compared with the situation where there was instruction. Uechi et al. [31] also evaluated women’s self-perception of their PFM contraction and its agreement with an assessor’s evaluation, revealing that most of the participants believed that they were correctly contracting their PFM; however, only 33% actually achieved a correct contraction. Most of the participants were not physically active and had never participated in PFM training. Another study examining the self-perception of PFM contraction was conducted by Vermandel et al. [32], who found that the belief that a correct PFM contraction was achieved was false in at least one of five postpartum women. Vermandel et al. employed a visual assessment of an inward movement of the perineum, whereas Uechi et al. [31] used the Modified Oxford Scale, and in our study we used an abdominal ultrasound examination. The different assessment techniques and the different populations (active women in our study, postpartum women in that of Vermandel et al.) are most probably the reason for the diverse results obtained.

Finally, our study emphasized the importance of PFM evaluation by a professional examiner who could educate women on how to correctly contract their PFM, as verbal instruction and their knowledge of their muscles may be insufficient. This is more important during movement, as voluntarily contraction is supposed to occur and prevent urinary incontinence.

### Limitations

In this study, we did not measure the amount of bladder displacement (in cm) via ultrasound, which might have presented the further evaluation of PFM function. Other limitations include this not being a blind study (e.g., the examiner performed all study phases), the small sample size, and the possible differences between the Pilates classes due to the different teachers.

## 5. Conclusions

Most women who performed Pilates exercises correctly contracted their PFM. Approximately 20% of the women were unable to do so, and most women did not perform a voluntary contraction before leg movement; nevertheless, proper instruction did improve their ability. Instruction during Pilates exercises slightly improved the women’s self-perception of a PFM contraction taking place during leg movement. Instruction regarding PFM contraction should be given during exercises, especially when intra-abdominal pressure increases. This improves correct PFM contraction and prevents the downward movement of the bladder. Specific training for women with PFM difficulties is effective in reducing urinary incontinence.

## Figures and Tables

**Table 1:** Demographic characteristics of participants according to research groups.

	Instruction Group (N = 22) M (±SD)	Control Group (N = 24) M (±SD)	*p* Value
Age (years)	50.50 (±7.75)	47.25 (±9.16)	0.14
Height (cm)	163.81 (±7.37)	163.33 (±6.98)	0.66
Weight (kg)	65.54 (±8.61)	64.62 (±8.14)	0.54
Body mass index (kg/m^2^)	24.43 (±2.91)	24.33 (±3.55)	0.79
No. of births	2.36 (±1.06)	2.41 (±1.03)	0.71

**Table 2:** Frequencies of pelvic floor muscles (PFM) contraction during leg movement with and without instruction.

	Instruction Group N = 22, N (%)	Control Group N = 24, N (%)	Total N = 46, N (%)
**Leg movement** *** without *** **verbal instruction for PFM contraction**			
Correct contraction	1 (5%)	1 (4%)	2 (4.35%)
Contraction and release	2 (9%)	0 (0%)	2 (4.35%)
No contraction	11 (50%)	17 (71%)	28 (60.87%)
Downward movement of bladder	8 (36%)	6 (25%)	14 (30.43%)
**Leg movement** *** with *** **verbal instruction for PFM contraction**			
Correct contraction	4 (18%)	8 (33%)	12 (26.09%)
Contraction and release	13 (59%)	7 (29%)	20 (43.48%)
No contraction	4 (18%)	5 (21%)	9 (19.57%)
Downward movement of bladder	1 (5%)	4 (17%)	5 (10.87%)

**Table 3:** Prevalence of the agreement between a subjective and objective examination of a pelvic floor muscles (PFM) contraction according to research groups.

	Instruction Group N = 22, N(%)	Control Group N = 24, N(%)	Total N = 46, N(%)
**PFM contraction (crook lying position** *** with *** **verbal instruction)**			
Agreement	18 (82%)	19 (79%)	37 (80.4%)
No agreement	4 (18%)	5 (21%)	9 (19.6%)
**Leg movement** *** without *** **verbal instruction for PFM contraction**			
Agreement	20 (91%)	17 (71%)	37 (80.4%)
No agreement	1 (5%)	7 (29%)	8 (17.4%)
Partial agreement	1 (5%)	0 (0)	1 (2.2%)
**Leg movement** *** with *** **verbal instruction for PFM contraction**			
Agreement	5 (23%)	13 (54%)	18 (39.1%)
No agreement	5 (23%)	7 (29%)	12 (26.1%)
Partial agreement	12 (55%)	4 (17%)	16 (34.8%)

## Data Availability

The data used and analyzed during the present study are available from the corresponding author on reasonable request.
